# OrthoSelect: a protocol for selecting orthologous groups in phylogenomics

**DOI:** 10.1186/1471-2105-10-219

**Published:** 2009-07-16

**Authors:** Fabian Schreiber, Kerstin Pick, Dirk Erpenbeck, Gert Wörheide, Burkhard Morgenstern

**Affiliations:** 1 Abteilung Bioinformatik, Institut für Mikrobiologie und Genetik, Georg-August-Universität Göttingen, Goldschmidtstr. 1, 37077 Göttingen, Germany; 2 Department für Geo- und Umweltwissenschaften, Ludwig-Maximilians-Universität, Richard-Wagner-Str. 10, 80333 München, Germany

## Abstract

**Background:**

Phylogenetic studies using expressed sequence tags (EST) are becoming a standard approach to answer evolutionary questions. Such studies are usually based on large sets of newly generated, unannotated, and error-prone EST sequences from different species. A first crucial step in EST-based phylogeny reconstruction is to identify groups of orthologous sequences. From these data sets, appropriate target genes are selected, and redundant sequences are eliminated to obtain suitable sequence sets as input data for tree-reconstruction software. Generating such data sets manually can be very time consuming. Thus, software tools are needed that carry out these steps automatically.

**Results:**

We developed a flexible and user-friendly software pipeline, running on desktop machines or computer clusters, that constructs data sets for phylogenomic analyses. It automatically searches assembled EST sequences against databases of orthologous groups (OG), assigns ESTs to these predefined OGs, translates the sequences into proteins, eliminates redundant sequences assigned to the same OG, creates multiple sequence alignments of identified orthologous sequences and offers the possibility to further process this alignment in a last step by excluding potentially homoplastic sites and selecting sufficiently conserved parts. Our software pipeline can be used as it is, but it can also be adapted by integrating additional external programs. This makes the pipeline useful for non-bioinformaticians as well as to bioinformatic experts. The software pipeline is especially designed for ESTs, but it can also handle protein sequences.

**Conclusion:**

OrthoSelect is a tool that produces orthologous gene alignments from assembled ESTs. Our tests show that OrthoSelect detects orthologs in EST libraries with high accuracy. In the absence of a gold standard for orthology prediction, we compared predictions by OrthoSelect to a manually created and published phylogenomic data set. Our tool was not only able to rebuild the data set with a specificity of 98%, but it detected four percent more orthologous sequences. Furthermore, the results OrthoSelect produces are in absolut agreement with the results of other programs, but our tool offers a significant speedup and additional functionality, e.g. handling of ESTs, computing sequence alignments, and refining them. To our knowledge, there is currently no fully automated and freely available tool for this purpose. Thus, OrthoSelect is a valuable tool for researchers in the field of phylogenomics who deal with large quantities of EST sequences. OrthoSelect is written in Perl and runs on Linux/Mac OS X. The tool can be downloaded at

## Background

DNA and protein sequences provide a wealth of information which is routinely used in phylogenetic studies. Traditionally, single genes or small groups of genes have been used to infer the phylogeny of a group of species under study. It has been shown, however, that molecular phylogenies based on single genes often lead to apparently conflicting tree hypotheses [1]. The combination of a large number of genes and species in genome-scale approaches for the reconstruction of phylogenies can be useful to overcome these difficulties [2]. This approach has been termed *phylogenomics *[3].

Since complete genome sequences are available only for a limited number of species, many phylogenomic studies rely on EST sequences. EST sequences are short (~200 – 800 bases), unedited, randomly selected single-pass reads from cDNA libraries that sample the diversity of genes expressed by an organism or tissue at a particular time under particular conditions. The relatively low cost and rapid generation of EST sequences can deliver insights into transcribed genes from a large number of taxa. Moreover, EST sequences contain a wealth of phylogenetic information. Several recent phylogenomic studies used EST sequences to generate large data matrices, e.g. [4-7]. Such studies start with the generation of EST libraries for a set of species. Overlapping EST sequences from single coding regions are then assembled into contigs and orthologous genes are identified as a basis for phylogenetic reconstruction. Homologous sequences are called orthologs if they were separated by a speciation event, as opposed to paralogous sequences, which were separated by a duplication event within the same species [8]. If the last speciation event predates the gene duplication event, homologous sequences are called inparalogs [9]. Orthologs are usually functionally conserved whereas paralogs tend to have different functions [10] and are less useful in phylogenetic studies. (because true genealogical relationships among taxa can only be reconstructed with great difficulty.) A typical protocol for detecting orthologs in phylogenomic studies should include (1) a similarity search using tools like BLAST [11], (2) a strategy to select a subset of hits returned by this search, (3) a criterion to identify sequences as potential orthologs, (4) a strategy for eliminating potential paralogs – in case several sequences from the same species have been assigned as potential orthologs to the same orthologous group.

Orthology assignment is a crucial prerequisite for phylogeny reconstruction as faulty assumptions about orthology – e.g. the inclusion of paralogs – can lead to an incorrect tree hypothesis [12]. Errors can result from similarity searches against non-specialized databases, e.g. NCBI's *nr *database, or from best-hit selection strategies such as *best reciprocal hit *[13] or *best triangular hit *that may lead to false positive orthology predictions. The similarity between a query and a database sequence stemming from a similarity search – expressed for example as a bit-score or expectation value (E-Value) – is usually taken as a criterion to predict an orthologous relationship. Since the results of these methods depend on the choice of a database and on the strategy to select sequences from similarity search hits, a more reliable protocol for ortholog predictions is needed.

Several databases and computational methods for predicting orthologs are available. Multi-species ortholog databases have been developed based on different sources of orthologous information. They include information about orthologous relationships between sequences. The OrthoMCL-DB database [14] and the KOG database [15] have been constructed from whole genome comparisons, HomoloGene [16] on the basis of synteny. HOVERGEN [17] and TreeFam [18] were constructed using the orthologous information from phylogenetic trees. Two of these databases, OrthoMCL-DB and KOG, explicitly define orthologous groups (OG) which can be used as a source for orthology assignment of unknown sequences using similarity searches.

Most computational methods to identify orthologs are based on either a phylogenetic analysis, or on *all-against-all *BLAST searches [19]. The former approach is computationally expensive and usually requires manual intervention. *All-against-all *approaches use every sequence from the input data set as a query for BLAST searches against sequences from the respective other species. This generates OGs based on some similarity measure, e.g. using all best reciprocal hits. These OGs can further be processed to merge, delete, or seperate overlapping groups using a clustering algorithm, as implemented in e.g. OrthoMCL [20] or Inparanoid [21]. Zhou and Landweber [22] developed BLASTO, a different computational method for orthology prediction by including information from an orthologous database. Other important aspects in data set construction for phylogenetic analysis on a large scale are (1) correct identification of open reading frames in ESTs and their translation, (2) careful selection of target genes to maximize the phylogenetic information, (3) elimination of redundant sequences, and (4) a refinement step to select conserved blocks and remove homoplasy from multiple sequence alignments.

Nowadays, data sets in phylogenomic studies can easily contain dozens of taxa and hundreds of genes [6]. The construction of data sets of that size for phylogenomic studies is time-consuming and can hardly be achieved manually. To the best of our knowledge, no software pipeline is currently available that performs the above steps automatically. Herein, we present a software pipeline, called OrthoSelect, to process clustered EST sequences automatically for phylogenomic studies. Our goal is to give both non-bioinformaticians and bioinformatic experts a useful framework to carry out analyses on a phylogenomic scale. It integrates publicly available bioinformatic tools and manages data processing and storage. Although the software pipeline is designed to automate the construction of data sets for phylogenomic studies, the user can evaluate intermediate results at any time of the analysis. OrthoSelect produces automatically calculated and post-processed alignments that can be used as input for common phylogenetic reconstruction software. In a large-scale study, we applied OrthoSelect to a data set from metazoan species consisting of > 950, 000 ESTs belonging to 71 taxa (unpublished data). In order to assess the quality of OrthoSelect predictions in relation to results obtained from other methods, we compared OrthoSelect to the manually created and published phylogenomic data set by Dunn et al. [6]. Since our tool offers an increased functionality compared to other tools for orthology prediction (e.g. OrthoMCL), our tests focus on the assignment of orthology only, and do not cover the correct translation of ESTs, gene selection, alignment computation, and alignment postprocessing.

### Implementation

**Figure 1:**
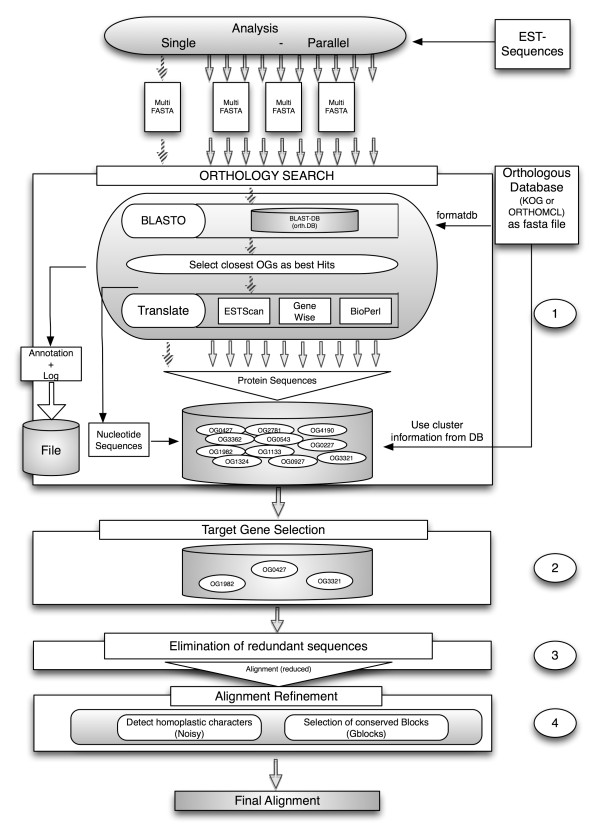
**Workflow of OrthoSelect**. The main workflow of the software pipeline to detect ortholog sequences in phylogenomic studies. Input are EST libraries and an ortholog database (either KOG or OrthoMCL) as multi-fasta files. The analysis comprises four parts. (1) The orthology detection – which can be performed on a single computer or a computer cluster – blasts each EST against the ortholog database, selects the closest ortholog group as the best hit and translates it and stored together with the nucleotide sequences in the corresponding OG. (2) Target genes can be selected. (3) The sequence most likely being an ortholog is selected by eliminating potential paralogs. (4) Informative alignment columns are selected to increase the phylogenetic signal.

Our software pipeline is written in *PERL *and uses BioPerl [23]. The main workflow is depicted in Figure 1. The entire analysis is guided by a configuration file and several *PERL *scripts. OrthoSelect can be run on a single desktop computer as well as on a computer cluster using a batch system, e.g. a Sun Grid Engine [24]. Required programs are *BLAST *for the similarity search, *ESTScan *[25] and *GeneWise *[26] for translating ESTs, and a software program for multiple sequence alignment. *ClustalW *and *MUSCLE *are needed for computing the pairwise sequence alignments. Our software supports multiple alignments computed by *MUSCLE *or *T-Coffee*, but it can easily be adapted to accept multiple alignments calculated by other programs. *Gblocks *[27], *Noisy *[28] and Aliscore [29] are used to select informative alignment columns. OrthoSelect offers the possibility to automatically download and install all missing required programs on the computer.

### Program outline

**Figure 2:**
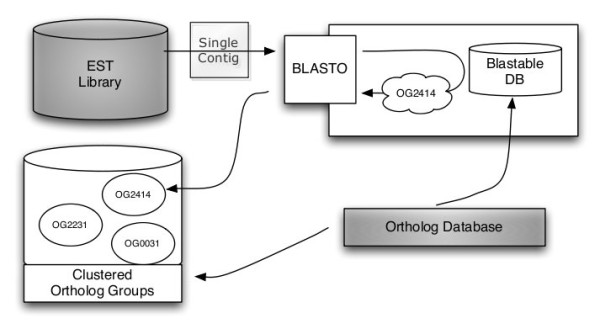
**Workflow of orthology assignment**. Workflow of our software pipeline. The two databases colored in green are to be supplied by the user. The ortholog database is converted into a BLAST database and clustered in ortholog groups. Each contig from the assembled EST library is assigned to the OG returned by a BLASTO search against the ortholog database.

In contrast to the above outlined methods for the identification of orthologs based on whole genome comparisons, we adopted an approach that compares EST sequences to predefined groups of orthologous genes. We developed a software pipeline that uses a reimplementation of BLASTO, an extension of BLAST that clusters BLAST hits using predefined orthologous groups from an ortholog database. Here, the similarity between a query sequence and an OG is defined as the mean E-value between the query and the sequences from the OG (see Figure 2). As input data, it takes a library of EST sequences together with a database of orthologous genes. We assume that the basic pre-processing steps such as end clipping and vector trimming have already been done and that the ESTs are already assembled into contigs. As a database of orthologs, either KOG or OrthoMCL-DB can be used.

Using the orthologous groups (OG) defined by KOG or OrthoMCL-DB as a basis, orthologous ESTs are detected by a similarity search of ESTs against the ortholog database and assigning them to the OGs using our reimplementation of BLASTO. The ESTs are then translated and stored. Redundant sequences within each OG are eliminated and an alignment of the remaining sequences is computed. In a last step, we use sophisticated post-processing methods to filter out non-informative or misleading information from the alignment (see Figure 1). The entire analysis is guided by a configuration file containing the main parameters and options for each external program.

### Orthology Detection

The first step of the software pipeline comprises the detection of potential orthologs in EST libraries (see Figure 1, Point 1). This is a critical step, because false ortholog assignments can lead to serious errors in the resulting phylogenetic tree. Orthologs are detected by searching an ortholog database – either KOG or OrthoMCL-DB – with a query EST using *blastx *and subsequently the resulting hits are clustered according to an algorithm similar to that used in BLASTO. A standard BLAST search returns a list of hits ordered by their significance. By contrast, BLASTO calculates similarity values between the query sequence and entire groups of orthologs (OGs).

In BLASTO, the similarity between a query *s *and a OG *g *is defined as the average similarity between *s *and all sequences in *g*. In our approach, we modified this measure of similarity. For a query *s *and a OG *g*, we consider only the subset *g' *⊂ *g *that contains the best hit from each species. This is to compensate the many paralogs present in KOG [30], and to ensure a high probability of the EST sequence being orthologous to the sequences in the corresponding OG. The similarity score for a query *s *and an OG *g *is then calculated as



where



Here, *E*_*i *_is the E-value of the BLAST alignment of *f*_*i *_with the query sequence *s *and |*g'*| the number of species in *g'*. Finally, every EST sequence *s *is assigned to those orthologous groups *g *with a similarity score *S*_*g*,*s *_above a given threshold. We allow multiple assignments of a single EST, because ESTs can represent domains rather than full genes, and they should be assigned to all OGs containing that domain (E.g. the OGs KOG0100, KOG0101, KOG0102 of KOG all contain the same Pfam domain *HSP70*). All ESTs assigned to the same OG are now potential orthologous. Redundant sequences will be removed later (see section *Eliminating Redundancies*).

### EST Translation

In the next step, potential coding regions in assembled EST sequences are detected and translated into proteins. By their nature, EST sequences often contain sequencing errors and may cover genes partially, only [31]. These errors result in e.g. reading frame shifts that make translation non-trivial. Several algorithms have been developed to overcome this problem. DIANA-EST [32] uses a combination of Artificial Neural Networks while ESTScan uses Hidden Markov Models. In contrast to this, DECODER [33] implements rule-based methods, and GeneWise uses a known protein as a template. In addition, combinations of these methods have been proposed to identify coding regions and to translate EST sequences correctly, e.g prot4EST [31]. We use a comparative approach of different well established programs for translation. Each EST is translated (using ESTScan, GeneWise, and a standard six-frame translation using BioPerl) and aligned to the best hit from the previous BLAST search using bl2seq [34]. The translated sequence with the lowest E-value is then chosen as the correctly translated sequence. This way, the probability of getting correctly translated ESTs is increased. Our goal was to fully automate the installation of all external programs. We did not include prot4EST since it requires additional programs and one of which is not freely available for download and therefore cannot be installed automatically.

### Taxon/Gene Sampling Strategy

After the assembled EST sequences were assigned to predefined orthologous groups (OG) and translated into proteins, the next step consists of the proper selection of OGs suitable for phylogenetic analysis. Since EST libraries represent snapshots of expressed genes, not every OG will contain EST sequences from all species under study; some OGs may contain too few sequences and do not contain sufficient information for further consideration. We do not require every OG to contain all sequences of interest. There is no consensus about the influence of missing genes on the resulting phylogeny [35]. No reliable criterion, which OGs should be used for phylogenetic inference exists. Our software offers two alternative ways of selecting OGs:

1. The user selects a subset of individual species under study. In this case, those OGs will be selected that contain at least one EST from each of the user-selected species.

2. The user defines *groups *of species (e.g. groups that are thought to be monophyletic). Our tool will then select those OGs that contain at least one EST sequence for each of the specified groups.

The idea of these two methods is to select the maximal biclique of a graph with the nodes consisting of the OGs and the taxa – in case of option 1 – or monophyla – in case of option 2 [36]. The selection of genes according to these who methods focusses on maximising the phylogenetic signal in the dataset (see Figure 1, Point 2).

### Eliminating Redundancies

Multiple divergent copies of the same gene and different levels of stringency during EST assembly can lead to a situation where OGs contain more than one sequence for each species (Depending on the size of the study, OGs can contain hundreds of sequences which makes manual elimination of redundant sequences impossible). It is also known that some of the orthologous groups contained in KOG contain not only orthologous genes but also paralogs [30]. In these cases, a fast and reliable method is needed to select the correct sequence for each species. We work with the assumption that a gene from one organism is often more similar to an orthologous gene from another organism than to paralogs from that organism. This seems plausible based on both the definition of orthology and the fact that orthologs typically retain the same function [10]. A scenario where a gene from one organism is more similar to a paralog rather than to its ortholog from another organism would require a considerable difference in the rate of paralog evolution [10]. Since this is more an exception than a rule and since OrthoSelect aims at the production of gene alignments containing only one sequence per species, we do not consider such cases.

**Figure 3:**
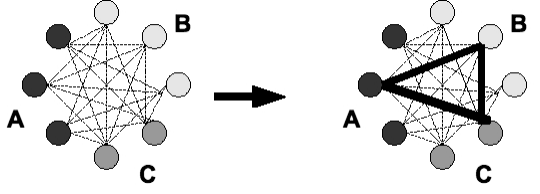
**Eliminating redundant sequences**. The figure shows how OrthoSelect eliminates redundant sequences. Here, we have an OG with three sequences from organism A and B and two sequences from organism C. All sequences are aligned in a pairwise manner to compute a distance matrix (left side). That sequence from an organism is selected that most often has the smallest distance to another organism, see section for details (right side).

All sequences belonging to the same OG are aligned in a pairwise manner to compute a distance matrix. Two types of distance matrices can be used to select the sequence from an organism that is most likely ortholog (see Figure 3):

1. An initial distance matrix as computed by alignment methods like *ClustalW *[37].

2. A normalized distance matrix selecting those sequences that have the highest percentage of matching positions in pairwise comparisons using *MUSCLE *[38,39].

The first option follows the idea that those sequences should be selected that optimize the alignment score in a global alignment. The second option takes into account that ESTs usually do not represent complete genes. Since a selection based on a standard distance matrix will penalize missing positions, longer paralogous sequences can be selected instead of shorter orthologous ones. The distance matrix used in the second option selects the sequence with the highest number of matching positions normalized by its length. The user can select one type of matrix to be used to eliminate redundant sequences (see Figure 1, Point 3). Based on that distance matrix, we want to select one sequence from each organism in such a way that the selected sequences are most probable to be ortholog to each other. Here, we use the following strategy: All sequences from one organisms are compared to all sequences from all other species. For each sequence *s *from a given species *S*, we count the number of species *S' *such that *s *has the shortest distance to a sequence from *S' *among all sequences from *S' *(if there are any such species *S'*). Formally, if the distance between sequences *s *and *s' *is denoted by *d*(*s, s'*), we count the number of species *S' *for which we have



For species *S*, we then select the sequence *s *for which this number is maximal (see Figure 3).

### Multiple Sequence Alignment

By default, the previously selected sequences are aligned using either *MUSCLE *or *T-Coffee *[40,41]. Other standard methods for multiple alignment can be used as well, e.g. *ProbCons *[42], *MAFFT *[43,44], *DIALIGN *[45,46] or *DIALIGN-TX *[47,48].

**Figure 4:**
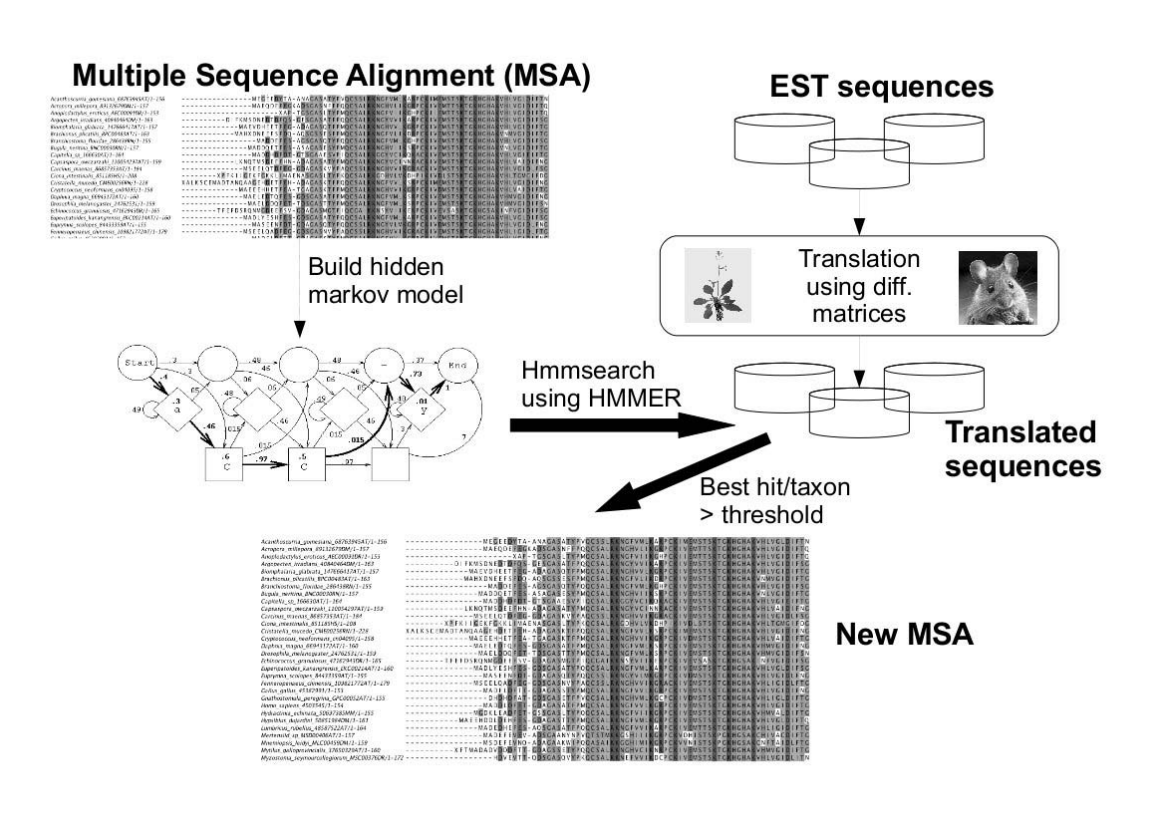
**Rebuilding the multiple sequence alignment**. The figure illustrates how OrthoSelect refines the multiple sequence alignments (MSA) created so far. Based on the MSA a hidden Markov Model (HMM) is build. Additionally, all EST libraries are translated using ESTScan with different matrices (ranging from *Arabidopsis thaliana *to *Homo sapiens*). The software *hmmsearch *from the HMMER package then used the HMM to search all translated sequences and selecting the best hit from each taxon above a given threshold. From these hits the new MSA is then computed

The computed alignments contain sequences that are most likely being orthologous given the data set. Nevertheless, there might be cases in which our comparative approach did not find the optimal translation (see section about EST Translation). To correct this, we use the software *hmmbuild *from the HMMER package to build profile hidden markov models (HMMs) from sequence alignments [49]. Additionally, the EST sequences from all taxa are translated using ESTScan. ESTScan is based on a HMM and was trained for species ranging from *Arabidopsis thaliana *to *Homo sapiens *by default. The translated sequence databases are then searched using hmmsearch from the HMMER package [50] and the HMM. The closest sequence from each taxon above a given threshold is taken as a hit. By this, we can find more similar as well as additional hits – hits that might have been overseen during the initial blastx search, because the EST sequence contained one or several frame shift errors. The workflow is depicted in Figure 4. The advantage of using a HMM is the possibility of finding that translated sequence that fits best to the whole existing alignment and not just to single sequences, as with standard Blast searches.

Once multiple alignments have been calculated for selected groups of ortholog EST sequences, these alignments can be further processed to exclude columns that are not suitable for phylogenetic analysis. Since not all parts of a gene evolve at the same rate, alignments typically contain highly conserved as well as less conserved sites. Alignment columns that are too conserved do not contain any phylogenetic signal. The same holds true for parts of the sequences that are too divergent to be correctly aligned. Another problem that confuses phylogenetic reconstruction is the presence of homoplasy caused by back- or parallel-mutation. Several programs have been developed to tackle these problems by automatically selecting sufficiently conserved blocks from alignments, for example *Gblocks *and *Aliscore*, or by eliminating potentially homoplastic sites, e.g. *Noisy*. *Gblocks*, *Aliscore*, and *Noisy *are incorporated in our software pipeline to allow a broad spectrum of alignment post-processing thereby increasing the accuracy of the subsequent phylogenetic analysis (see Figure 1, Point 4). Furthermore, alignments processed by *Gblocks *can be further filtered by discarding too short sequences from the alignment (e.g. sequences with > 50% missing characters).

## Results and Discussion

OrthoSelect is the first fully automated and freely available tool that covers the whole process of selecting orthologs from EST libraries to output orthologous gene alignments that can be used to build phylogenies. In the absence of a gold standard for benchmarking of orthology prediction and in order to evaluate the performance of our program, we designed the following tests: First, OrthoSelect was compared to the best-hit selection strategy using a set of sequences from JGI with KOG-annotations. Second, we evaluated the performance compared to the KOG database by re-annotating (re-assinging) ortholog database sequences. In the third and most powerful test we compared OrthoSelect tool to a manually created and published phylogenomic data set. In this context, we also compared our tool with OrthoMCL.

### OrthoSelect vs. Best-hit selection strategy

To evaluate the performance of our software pipeline and the best-hit selection strategy regarding correct orthology assignment, we used a data set comprised of transcribed genes and annotation files from 4 different species as shown in Table 1. The best-hit selection strategy assigns the query sequence to that OG the best hit belongs to. As ortholog database, we used KOG. The annotation files contain KOG classification and therewith the functional annotation for each sequence. Sequences and annotations were downloaded from the Department of Energy Joint Genome Institute (JGI) [51]. Since OrthoSelect makes annotations by assinging sequences to OGs of KOG, we considered an assignment of a sequence to an OG to be correct if it matches the KOG classification provided by JGI. To evaluate the performance of our classification system, we calculated for each species and an E-value cut-off of 1*e *- 10 the ratio of correctly assigned OGs, i.e. the number of correctly assigned sequences divided by the number of assigned sequences. Table 2 shows the result of the analysis. Our software pipeline reaches a correct assignment rate of ~93%, whereas the best-hit selection strategy assigns the sequences in ~79% of the cases to the correct OG. OrthoSelect outperforms the best-hit selection strategy and its very high rate of correct ortholog prediction should provide a good basis for subsequent phylogenetic analyses.

**Table 1:** Species used.

*Species*	*Sequences*	*KOG Classifications*
*Daphnia pulex*	30940	15806
*Ostreococcus tauri*	7725	4733
*Trichoderma virens*	11643	6879
*Xenopus tropicalis*	27916	27617

The table shows species that we used in our test runs along with the number of sequences from each sequence and the corresponding KOG classifications.

**Table 2:** Results from orthology assignment: OrthoSelect vs. Best-hit selection strategy.

*Species*	*Predictions*	*OrthoSelect*	*Best-hit strategy*
*Daphnia pulex*	12696	98%	86%
*Ostreococcus tauri*	4742	91%	76%
*Trichoderma virens*	5886	99%	87%
*Xenopus tropicalis*	18556	84%	69%

The table shows species that we used in our test runs along with the number of predictions and percentage of correct predictions made by OrthoSelect and the best-hit selection strategy respectively.

### OrthoSelect vs. KOG

In absence of a reference dataset for orthology prediction and due to the fact that our tool is mainly focused on the automation of a process rather than being a completely new method for orthology prediction, we compared OrthoSelect to the KOG database by re-annotating (re-assinging) ortholog database sequences. We performed the following: 5000 sequences were randomly chosen and masked out from the ortholog database. The remaining sequences were converted into a blastable database. We then ran OrthoSelect using each of the 5000 sequences as a query sequence against the masked database. Assuming the original ortholog group assignment in the ortholog database represents the correct orthology relation, we calculated in how many cases our orthology assignment matched the original assignment. We could assign the query sequences in 92% of the cases to the correct ortholog group.

### OrthoSelect vs. manually created data set by Dunn et al

The goal of our tool is to automate the process of constructing data sets that can be used for subsequent phylogenetic analyses. To test our tool regarding this, we selected Dunn et al.'s data set (hereafter referred to as reference data set) published in *Nature *[6].

This data set consists of newly sequenced ESTs as well as publicy available ESTs and protein sequences, and has been generated using all-vs.-all BLAST searches, protein translations using prot4EST, grouping of the sequences into orthologous groups using TribeMCL [52] as well as manual curation and tree reconciliation (see [6] for more details).

The reference data set as well as the single EST and protein sequences were either downloaded from publicly available sources or provided by Casey Dunn. The initial data set consisted of 150 genes and 77 taxa. In order to guarantee comparable results, we mapped each sequence from each gene to the KOG database using the best BLAST-hit. Only genes where all sequences could be mapped to the same KOG were further considered. This led to a considerable decrease in the number of genes. Since some taxa were not available for download, we ended up with 70 out of the 77 taxa Dunn et al. initially used.

For prediction of orthologous sequences, we denote a true positive as a correctly predicted ortholog, a false positive as an incorrectly predicted ortholog, and a false negative as an overlooked sequence. To be more precise, we use the following measures of performance:

• *Taxon is present in both alignments: *If the percentage identity of both sequences is above a threshold (≥ 95%), the sequences are regarded as being equal and counted as a true positive. Else, both sequences are aligned to a hidden markov model (HMM) build from the alignment of the corresponding orthologous group (OG) using hmmsearch from the HMMER package. If the OrthoSelect sequence is closer to the HMM, it will be counted as a true positive, and otherwise it will be counted as a false positive.

• *Taxon is present in the reference alignment, but not in the OrthoSelect alignment: *It will be counted as a false negative.

• *Taxon is present in the OrthoSelect alignment, but not in the reference data set: *The sequence is aligned to the HMM of that OG. If it shows significant similarity, it will be counted as a true positive, and otherwise as a false positive.

Furthermore, we use the following formula to measure the specificity of our results:

• *Specificity:*



We get the following results (see also Table 3): With respect to the reference data set, our tool receives a specificity of 98%. This means that the predictions about orthology our tool makes are almost always true and almost all orthologous sequences contained in the original reference data set could be found. The number of false predictions is considerably small. Although we missed 8% of the orthologous sequences, we could find additional hits for 270 sequences. 268 of those additional sequences showed significant similarity to the rest of the alignment and were counted as true positives. 2 sequences were falsely predicted as being orthologous. This equals an increase of +4% of orthologous sequences. Compared to the reference data set, we can make the following statements: Our tool selects orthologous sequences from EST libraries and other sources with very high accuracy. OrthoSelect correctly translates the sequences and receives a higher specificity by finding more true positives. In phylogenomics, the use of EST data can result in data matrices – where the rows are genes and the columns are taxa or vice versa – with most of the cells being empty. Although there is no consensus about the impact of missing sequences on the resulting phylogeny, the additionally found sequences will have a beneficial effect.

**Table 3:** Results from orthology assignment: OrthoSelect vs. reference data set

*Value*	*OrthoSelect*
*Specificity*	98%
*Cases where OrthoSelect found better sequences*	63%
*Number of additional sequences found*	270
*Number of additional sequences found (good)*	268
*Number of additional sequences found (bad)*	2
*Number of sequences missed*	197
*Ratio of additional/missed sequences*	+4%

### OrthoSelect vs. OrthoMCL

**Figure 5:**
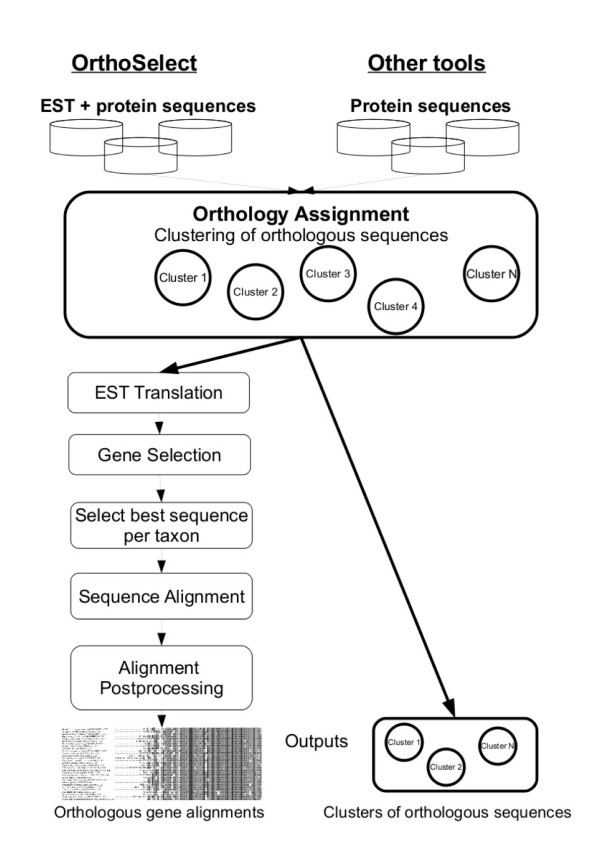
**Overview of functionality of OrthoSelect compared to other tools**. The figure illustrates the differences in functionality between OrthoSelect and other tool for orthology prediction. Both approaches have in common that they build clusters of orthologous sequences. Moreover, OrthoSelect can handle EST sequences and correctly translate them and further processes these clusters to select only one sequence per taxon, compute sequence alignments and refine them. In contrast to the other tools, OrthoSelect outputs orthologous gene alignments that can be directly used the subsequent phylogenetic analysis.

In order to further assess the performance of OrthoSelect, we compared it with OrthoMCL, another tool for orthology prediction. OrthoMCL takes a set of sequences and clusters them into groups of orthologous and inparalogous sequences. In contrast to OrthoSelect, OrthoMCL only handles protein sequences and produces clusters of orthologous sequences rather than multiple sequence alignments (see Figure 5). These generated clusters can contain considerably more than one sequence per taxon, and subsequently build multiple sequence alignments would not be comparable to the ones produced by OrthoSelect and Dunn et al. Nevertheless, we are interested in the performance of our tool compared to OrthoMCL. The previous test revealed that clustering algorithms of OrthoSelect and the method by Dunn et al. perform similarly. To check if clusters build by OrthoMCL are in agreement with the OrthoSelect clusters and thus with the Dunn clusters, we used the following 6 taxa: *Cryptococcus neoformans*, *Drosophila melanogaster*, *Gallus gallus*, *Homo sapiens*, *Saccharomyces cerevisiae*, and *Suberites domuncula*. The dataset has been reduced to include only protein sequences, because OrthoMCL deals with protein sequences, only.

For each of the 60 previously compared gene clusters (see previous section), we checked whether OrthoMCL assigns sequences from the 6 taxa to the same OrthoMCL cluster or not. The results were, that all sequences belonging to the same alignment have been clustered together by OrthoMCL. This means that the clustering algorithm of all methods produce similar results and converge.

Besides the additional functionality of OrthoSelect as compared to OrthoMCL and its usability for EST sequences, it is also much faster. It took OrthoMCL 24 hours to analyse the data set of 55.646 sequences. In contrast, our tool analysed the 1.000.000 sequences Dunn et al. used in about 6 hours.

## Conclusion

OrthoSelect is a tool for finding ortholog groups in EST databases. It can be used by either installing it locally or via the OrthoSelect web server [53]. It automatically searches assembled EST sequences against databases of ortholog groups (OG), assigns ESTs to these predefined OGs, translates the sequences into proteins, eliminates redundant sequences assigned to the same OG, creates multiple sequence alignments of identified ortholog sequences and offers the possibility to further process these alignments in a last step. OrthoSelect performes better than the best-hit selection strategy and shows reliable results in re-annotating database member sequences of OrthoMCL-DB and KOG. Most importantly, we showed that our tool produces high quality data sets such as Dunn et al's data set, but with more selected sequences and therefore less missing data in the alignments. Furthermore, the results our tool produces are in absolut agreement with the results of OrthoMCL, but OrthoSelect offers additional funcionality, e.g. handling with EST sequences, computing sequences alignments, and refining them. Our method also showed a significant speedup in comparison to OrthoMCL. Correct orthology assignment is an important prerequisite for the construction of reliable data sets and OrthoSelect is capable of producing them. This makes a OrthoSelect a valuable tool for researchers dealing with large EST libraries focussing on constructing data sets for phylogenetic reconstructions. The tool can be downloaded at  or the web server accessed without local installation at .

## Availability and requirements

**Project name**: OrthoSelect

**Project home page**: 

Operating system: Mac OS X, Linux

Programming language: Perl

Other requirements: BioPerl, BLAST, ESTScan, GeneWise, Clustalw, Muscle or T-Coffee, HMMER, Gblocks, Aliscore or Noisy

**License**: GNU GPL

**Restrictions**: none

## Authors' contributions

FS developed, implemented, tested OrthoSelect, and wrote the manuscript. KP and DE evaluated the performance and usability of OrthoSelect and revised the manuscript. GW conceived and co-supervised the project, provided resources and revised the manuscript. BM co-supervised the project, provided resources and participated in writing of the manuscript. All authors read and approved the final manuscript.
